# Laparoscopic Cholecystectomy in Patients With Situs Inversus Totalis: Literature Review of Two Patients

**DOI:** 10.5812/ircmj.4806

**Published:** 2012-12-06

**Authors:** Ismail Demiryilmaz, Ismayil Yilmaz, Yavuz Albayrak, Kemal Peker, Atalay Sahin, Nurdan Sekban

**Affiliations:** 1 General Surgery Department, Ibni Sina Hospital, Kayseri, Turkey; 2 Department of Surgery, Medical School, Erzincan University, Erzincan, Turkey; 3 General Surgery Department, Regional Education and Research Hospital, Erzurum, Turkey; 4 Department of Thoracic Surgery, Medical School, Dicle University, Diyarbakir, Turkey; 5 Anesthesiology and Critical Care Department, Ibni Sina Hospital, Kayseri, Turkey

**Keywords:** Situs Inversus Totalis, Laparoscopy, Cholecystectomy

## Abstract

Situs inversus totalis is a rare condition, which presents difficulties in diagnosis and treatment of gallstones due to the reversal location of abdominal organs. In this article we present 2 cases of women in age of 51 and 55 years with situs inversus totalis and gallstones. There are described the clinical and imaging features, also the laparoscopic surgery with the difficulties encountered by right handed surgeon. In patients with situs inversus totalis, laparoscopic surgery may be performed safely by a surgeon with experience.

## 1. Introduction

Situs inversus totalis (SIT) is a rare anomaly in which the thoracal and abdominal organs are reversely rotated on the mirror view (1). In the partial type, it is known as dextrocardia, the heart is located on the right side. However in the complete form, the liver and gall bladder are on the left and the stomach and spleen on the right. The patients with SIT are generally asymptomatic and maintain a normal lifetime. The reported cases are not so many in the literature. Laparoscopic surgery, was first performed by Mauret in 1987, and became the standard treatment for symptomatic cholelithiasis in patients. Nowadays, it is safe and feasible for better cosmetic results; short-term hospitalization and less invasiveness (2, 3). We studied 2 cases of successful multiport laparoscopic cholecystectomy with SIT and describe in our practice.

## 2. Case Reports

### 2.1. Case 1

A 55-year-old woman with known SIT complained from dyspepsia, distension and some pain in the right upper quadrant for 2 years. She was admitted to our hospital due to the history of intermittent epigastric discomfort and the aching pain radiating to the left upper quadrant for the last 2 months. the tenderness was found in deep palpation during physical examination. A chest X-ray and abdominal ultrasonography confirmed dextrocardia and the reversal of her organs. Abdominal ultrasonography and computed tomography showed some calculi in the gall bladder and that the liver was located in the left upper quadrant, whereas the spleen and stomach were located on the right side. The laboratory data were within normal range.

### 2.2. Case 2

A 51-year-old woman had occasionally dyspepsia, dyscomfort and some pain in the left upper quadrant within the last 6 months. She was admitted to emergency department due to her complaint aggravated within the last 2 days. The tenderness in physical examination, to epigastric discomfort and the aching pain radiating to the left upper quadrant were reported. A chest X-ray and abdominal ultrasonography confirmed dextrocardia and the reversal of her organs. Abdominal ultrasonography and computed tomography showed choledocolythiasis and a thick wall (4mm). The laboratory tests were within normal range except leucocytosis. Her chest x-ray and abdominal tomography scan were shown in Figure 1 and 2.

### 2.3. Operative Technique

In both cases, the primary surgeon and the camera assistant stood on the right side of the patient and the monitor on the left. An incision was made below the umblicus. Abdominal cavity was insuffled and a 10 mm trocar was introduced into the abdomen through this hole. Then using a 0º camera, 1 and 0.5 cm incision below xyphoid were used 5 and 10 cm medial to the left anterior iliac spine in order to enter the abdomen. Intra abdominal pressure was maintained 14 mm Hg. Dissection at Calot’s triangle in these patients was started by using dissectors through the left side trocar with the right hand as the surgeon used the right hand in normal patients. Cystic artery in these patients lies down posteriorly because of the clockwise rotation. The Cystic duct and artery were found, clipped and divided. The laparoscopy was completed successfully in 80 minutes for the first patient and 50 minutes for the second. The patients were discharged the next day. No complication was noted at the follow up.

## 3. Discussion

Situs inversus totalis is a rare congenital disorder, found in 1:5000-20000 of the population (1, 4). This anomaly may involve thoracic organs or abdominal viscera (situs inversus parsialis) or both (situs inversus totalis) (5). No racial or gender predilection is presented (6). The patients have a normal lifetime unless cardiac defects are severe. The extrahepatic biliary, venous or arterial anomalies are the same as those in normal population. In cases of situs inversus partialis, less common, some anomalies of biliary tractus and vascular structures. It is suggested to perform intraoperative cholangiography and to turn to open surgery in such cases (7). Open surgery was asserted to be more safe in patents with SIT in the study (8). The incidence gall stones and followed by acute cholecystitis is the same in normal population (4). Difficulty can be encountered in diagnosis due to the reversal locations of abdominal viscera (9). Some authors suggest the prophylactic appendectomy during laparoscopy for any purpose in patients with SIT(10). SIT can be easily diagnosed after chest x-ray, electrocardiogram, abdominal US, computerized tomography or barium swallow (11). We noticed SIT after CXR and abdominal US in one of our cases. The pain was on epigastric area or left upper quadrant and radiating to the left shoulder. It is important to be aware of the presence of the pain in unusual place (6, 7, 9). The pain in the right side is also possible in 10% of patients (7). Laparoscopic cholecystectomy (LC) has become the gold standard for treating symptomatic cholelithiasis (2, 3). Laparoscopic surgery has gained reputation worldwide owing to new instruments and growing experience in surgery (12). Indications of laparoscopic cholecystectomy are the same as those of open surgery but contraindications vary according to the surgeon’s skills (13). SIT is shown to be not a contraindication to laparoscopy in many reports (4, 7). The fact is that in laparoscopy in patients with SIT, everything is reversal on the mirror. The right handed surgeons can use their right hands during dissection of Calot’ s triangle through the left trocar since it is difficult to use the left hands(14). At this time the cystic artery, lying posteriorly, can be easily dissected by using the trocar below xiphoid for retraction of Hartmann’s pouch forward and backward. The Clips are pushed through this trocar. Variations in intra-abdominal structures and changing of cystic artery location are overcame by different positions of the abdominal ports in laparoscopic cholecystectomy.

## 4. Conclusion

SIT is a condition with left-right reversal of the viscera. Laparoscopic surgical procedures can easily performed with modification of the procedure. The right handed surgeons should give careful attention to anatomic variations during surgery, especially at the dissection of Calot’s triangle. A 30˚ camera has better view and it is used as possible. The improper sings during the examination and unusual image labeling before surgery should be checked in order to lessen complications related to SIT. It also prevents medico-legal implementations.
